# Prospective associations of appetitive traits at 3 and 12 months of age with body mass index and weight gain in the first 2 years of life

**DOI:** 10.1186/s12887-015-0467-8

**Published:** 2015-10-12

**Authors:** Phaik Ling Quah, Yiong Huak Chan, Izzuddin M. Aris, Wei Wei Pang, Jia Ying Toh, Mya Thway Tint, Birit FP Broekman, Seang Mei Saw, Kenneth Kwek, Keith M. Godfrey, Peter D. Gluckman, Yap Seng Chong, Michael J. Meaney, Fabian KP Yap, Rob M. van Dam, Yung Seng Lee, Mary FF Chong, on behalf of the GUSTO study group

**Affiliations:** Singapore Institute for Clinical Sciences (SICS), Agency for Science, Technology and Research (A*STAR), Singapore, Singapore; Department of Biostatistics, Yong Loo Lin School of Medicine, National University of Singapore, Singapore, Singapore; Department of Pediatrics, Yong Loo Lin School of Medicine, National University of Singapore and National University Health System, Singapore, Singapore; Department of Obstetrics & Gynaecology, Yong Loo Lin School of Medicine, National University of Singapore, Singapore, Singapore; Department of Psychological Medicine, National University Hospital, Singapore, Singapore; Saw Swee Hock School of Public Health, National University of Singapore, Singapore, Singapore; Department of Maternal Fetal Medicine, KK Women’s and Children’s Hospital (KKH), Singapore, Singapore; MRC Lifecourse Epidemiology Unit & NIHR Southampton Biomedical Research Centre, University of Southampton & University Hospital Southampton NHS Foundation Trust, Southampton, UK; Liggins Institute, University of Auckland, Auckland, New Zealand; Department of Psychiatry and Neurology & Neurosurgery, Douglas Mental Health University Institute, McGill University, Montréal, Canada; Department of Paediatric Endocrinology, KK Women’s and Children’s Hospital (KKH), Singapore, Singapore; Duke-NUS Graduate Medical School (GMS), Singapore, Singapore; Clinical Nutrition Research Center, Singapore Institute for Clinical Sciences (SICS), Agency for Science, Technology and Research (A*STAR), Singapore, Singapore; Singapore Institute for Clinical Sciences, Brenner Centre for Molecular Medicine, 30 Medical Drive, Singapore, 117609 Singapore

**Keywords:** Appetitive traits, Weight, Weight gain, BEBQ, CEBQ, BMI

## Abstract

**Background:**

Appetitive traits in childhood such as food responsiveness and enjoyment of food have been associated with body mass index (BMI) in later childhood. However, data on appetitive traits during infancy in relation to BMI in later childhood are sparse. We aimed to relate appetitive traits in infancy to subsequent BMI and weight gain up to 24 months of age.

**Methods:**

Data of 210 infants from the Singapore GUSTO mother-offspring cohort was obtained. The Baby Eating Behavior Questionnaire (BEBQ) and the Child Eating Behavior Questionnaire (CEBQ) were administered to mothers when their offspring were aged 3 and 12 months respectively. Height and weight of offspring were measured at ages 3, 6, 9,12,15,18 and 24 months. The association of appetitive traits with both BMI z-score and weight gain were evaluated using multivariate linear regression.

**Results:**

Food responsiveness at 3 months was associated with higher BMI from 6 months up to 15 months of age (*p* < 0.01) and with greater weight gain between 3 and 6 months of age (*p* = 0.012). Slowness in eating and satiety responsiveness at 3 months was significantly associated with lower BMI at 6 months (*p* < 0.01) and with less weight gain between 3 to 6 months of age (*p* = 0.034). None of the appetitive traits at 12 months were significantly associated with BMI or weight gain over any time period.

**Conclusion:**

Early assessment of appetitive traits at 3 months of age but not at 12 months of age was associated with BMI and weight gain over the first two years of life.

**Trial registration:**

Clinical Trials identifier NCT01174875

**Electronic supplementary material:**

The online version of this article (doi:10.1186/s12887-015-0467-8) contains supplementary material, which is available to authorized users.

## Background

Eating behavior has been associated with differences in body weight [1–4] in children. It has been reported that obese children tend to eat faster [1], and appear to display an impaired satiety signal as they fail to show the normal pattern of eating deceleration toward the end of a meal [2]. In contrast, children who are underweight are often described as fussy or slow eaters, and appear less interested in food [3, 5].

A widely used tool to assess individual variability in children’s eating behaviors [6, 7] is the Child Eating Behavior Questionnaire (CEBQ). This parent-reported questionnaire has been designed to measure appetite traits such as food responsiveness, enjoyment of food and satiety responsiveness in children as young as 1 years of age [8, 9]. Cross-sectional studies have reported increased satiety responsiveness to be associated with lower BMI, and both enjoyment of food and food responsiveness to be associated with higher BMI in children aged 3 to 13 years [10–13]. A longitudinal study using CEBQ has reported that satiety responsiveness at 2 years was inversely associated with energy intake and BMI z-score of children at 4 years of age while food responsiveness and enjoyment of food was not associated with either outcome [14].

While several studies have supported rapid weight gain in infancy being associated with greater risk of obesity in childhood and adulthood [15–17], recent studies show that this relationship is complex, with suggestions that much of the variance in weight gain in infancy maybe explained by lean rather than fat mass ([18, 19]). Whether this association is mediated by appetitive traits during infancy is still unclear and inconclusive.

On one hand, evidence from some studies suggests that conceptually similar eating behaviors that lead to obesity in children were also present during infancy [20, 21]. For example, infants that suckled more rapidly during feedings at 2 and 4 weeks had greater skinfold thicknesses and BMI at 2 years of age [20], and infants who often emptied bottles during the first 6 months of life were associated with excess weight between 6 to 12 months of age [22]. On the other hand, Wright et al. has observed a lack of association between a measure of appetite (eating avidity) and adiposity at 7 years of age and Svensson et al. found no association between child appetitive traits and BMI in children aged between 1–6 years old [9].

The Baby Eating Behavior Questionnaire (BEBQ) was recently developed as an infant version of the CEBQ to address the need for a psychometric measure of infant appetite. It characterizes dimensions of feeding behaviors when infants are still exclusively fed milk [23]. To date, only two longitudinal studies have been published using the BEBQ, both from the Gemini population-based twin cohort study in the United Kingdom. The first study reported appetitive traits such as enjoyment of food, food responsiveness, satiety responsiveness at 3 months to be prospectively associated with weight at 9 months of age as well as weight gain between 3 to 15 months of age [24]. The second study found that within-pair analyses, siblings with higher food responsiveness and lower satiety responsiveness had an increase in weight gain from 3 up to 15 months of age [25].

Despite the current literature, particular gaps in this area of research persist. Firstly, most studies to date are cross-sectional and there are limited longitudinal studies on the relationship between a child’s early appetitive traits and their weights or body mass index. Secondly, there are no known studies comparing the measurement of appetite traits at 3 months using BEBQ and at 12 months using CEBQ with childhood body mass indices and weight gain, at least in the first two years of life. There is increasing evidence that the first few months of life, until 3 months, is a critical period for preventing childhood obesity [26, 27]. This suggests a need for more studies to support the usage of the BEBQ as a possible tool for predicting childhood weight gain.

In this study, we aim to address the aforementioned gaps by comparing the prospective associations between appetitive traits measured at 3 and 12 months (using the BEBQ and CEBQ respectively), and BMI and weight gain from 3 months up to 24 months of age by using data from the Growing Up in Singapore Towards Healthy Outcomes (GUSTO) mother-offspring cohort.

## Methods

### Participants

We analyzed data from the GUSTO study, a mother-offspring cohort study involving detailed assessments of the characteristics of pregnant women and their offspring starting from the first trimester of pregnancy [28]. The primary objective of the study is to investigate the effect of early life events on the risk of health outcomes later in life. Participants were pregnant women receiving first trimester antenatal care from two major public maternity units in Singapore, the KK Women’s and Children’s Hospital (KKH) and the National University Hospital (NUH). The participants were recruited between June 2009 to September 2010 with the criteria that they had to be Singapore citizens or permanent residents who were delivering at either hospital and had the intentions to reside in Singapore for the next 5 years. Participants also had to be of Chinese, Malay or Indian ethnicity with a homogenous parental ethnic background and had to be willing to donate birth tissues including cord, placenta and cord blood after the delivery. Mothers receiving chemotherapy, psychotropic drugs or who had type I diabetes mellitus were excluded. Written informed consents were obtained from all participants [28]. More details are published in previous studies about the cohort [28]. The current study which is part of the GUSTO study was approved by the National Health Care Group Domain Specific Review Board and the Sing Health Centralized Institutional Review Board.

### Infant and maternal characteristics

Data on maternal ethnicity, age and education level were collected from participants during recruitment. Information about smoking during pregnancy and pregnancy BMI was collected at a clinic visit at 26–28 weeks gestation. Information on birth weight, gestational age, infant gender, and birth order was ascertained from birth records, and infant milk feeding data from infancy questionnaires administered at 3 weeks, 3 months and 6 months. At 3–18 months of age infant weight was measured to the nearest gram (g) (SECA 334 Corp. Hamburg, Germany) while the weight of toddlers at 24 months was measured to the nearest kilograms (kg) using calibrated scales (SECA 813 Corp. Hamburg, Germany). Recumbent infant crown-heel length was measured using an infant mat when the infant was 3–18 months of age (SECA 210 mobile measuring mat); the child’s standing height at age 24 months was measured using a stadiometer (SECA 213 Mobile Stadiometer). All measurements were taken by trained staff during either clinic visits or home visits. Both length and height were measured to the nearest 0.1 cm. For reliability, height and weight measurements were averaged from duplicate values.

### Appetitive traits

Appetitive traits were measured using the self-administered BEBQ [23] and CEBQ [8] questionnaires. The BEBQ was handed out to mothers during the 3-month post-partum home visit and collected at the end of the visit. The CEBQ was mailed out prior to the 12-month visit and collected during the 12 month home visit.

The BEBQ relates to a period of exclusive milk feeding [23], while the CEBQ relates to a period in which feeding was predominantly on solids [8]. Each item on the questionnaires was answered using a five-point Likert frequency scale (1 = never, 2 = rarely, 3 = sometimes, 4 = often and 5 = always). Factor analysis was performed to analyze the underlying structure of the questionnaire and to determine whether the structure was similar to the original BEBQ and CEBQ. Principal component analysis (PCA) with Varimax normalized rotation was run on all items of the BEBQ and CEBQ. Questions with reverse scales were first reverse scored, and a factor loading cut-off of 0.5 was applied before running the factor analysis. The 18- item, 4-factor original BEBQ resulted in a 17-item, 3-factor model after factor analysis in this study. Satiety responsiveness and slowness in eating items from the original model loaded onto the same factor and were thus combined into one subscale termed slowness in eating and satiety responsiveness (Additional file 1: Table S1). This observation mirrors previous studies that have demonstrated a similar combined subscale, but in the CEBQ [12, 29–31].

Factor analyses of the 35- item, 8-factor original CEBQ resulted in a 30-item, 7-factor model in this study. Items of the original enjoyment of food and food fussiness subscale loaded into the same factor and were thus combined into one subscale termed enjoyment of food (Additional file 2: Table S2). This could be due to the cultural differences in our population influencing the interpretation of the CEBQ items and is not dissimilar to studies in Malaysia [32] and Sweden [9]which revealed a nine and seven-factor structure respectively. To enable appetitive traits captured at both time points in our study to be comparable, we chose to focus our analyses on the subscales that are found in both the BEBQ and CEBQ. These are the food approach appetitive traits: food responsiveness and enjoyment of food and the food avoidant appetitive traits: slowness in eating and satiety responsiveness.

Sensitivity tests were conducted to ensure that the subscales generated were stable with imputation of missing items from the questionnaires. These were done by imputing a maximum of 3 missing items with various computations: the lowest item score, the highest item score, the mean score or randomly generated scores. These computations were compared with the initial BEBQ and CEBQ dataset without imputations. Factor analysis results showed good consistency between the factor structures of the BEBQ and CEBQ with and without imputed scores. Subsequently, up to a maximum of three missing items were imputed using mean values.

The internal reliability coefficients (Cronbach’s alpha) were calculated for each subscale of the BEBQ and CEBQ. The coefficients ranged from 0.6-0.9, which indicated a moderate to good internal reliability of the subscales of both questionnaires in this cohort (Additional file 1: Table S1 and Additional file 2: Table S2).

### Statistical analysis

For our analyses, we excluded infants born with a low birth weight and preterm born infants. Infants with low birth weight were defined as birth weight below 2500 g and preterm birth as the delivery of a live infant before 37 weeks of gestation.

Gender and postnatal age adjusted BMI z-score in this study were calculated according to the WHO 2006 Child growth standards [33]. The general linear model was then used to analyze the associations between the appetitive traits at 3 or 12 months of age (independent variable) and BMI z-score (dependent variable) at 3, 6, 9, 12, 15, 18 and 24 months of age. Potential confounders included into the model were BMI z-score at birth, gestational age, birth order, infant milk feeding patterns up to 6 months of age, mother’s nationality, mother’s education, mother’s age, smoking during pregnancy and BMI at 26 weeks of pregnancy.

Associations with weight gain from 3 months were also examined. Weight gain was measured by conditional BMI z-score change at intervals of 3 months up till 24 months (e.g. 3–6 months of age, 6–9 months of age). BMI z-scores at each successive time point, conditional on previous BMI z-score was calculated by saving the residuals from linear regression models of BMI z–scores at each successive time point versus BMI z-score at the earlier time point [34, 35].

## Results

### Characteristics of participants

Out of 3751 families screened, of which 2034 met eligibility criteria, 1247 women (response rate 61.3 %) were recruited [28], 368 were excluded from the final study as they were either dropouts from the study or pre-term infants, low birth weight infants, infants who had neonatal complications, twins, or infants conceived via *in vitro* fertilization; these conditions are known to influence the postnatal growth [36–38]. Among the remaining 879 participants, the percentage who completed BEBQs and CEBQs (administered in English) were 45.8 % (403/879), and 36.4 % (320/879) respectively. In total, 23.9 % (210/879) of all participants completed both the BEBQ and CEBQ.

Characteristics of participants who completed both questionnaires were broadly similar to those who completed either one questionnaire and those who did not complete any questionnaire, except those who completed both questionnaires tended to be older, belonged to the Chinese ethnic group, had an educational level up to university level or above and who breastfed their infants (Table 1).

**Table 1:** Characteristics of participants who completed either one questionnaire, compared to those who did not complete any questionnaire and those who completed both questionnaires

Characteristics	Completed both	Completed either one questionnaire	Did not complete any	*p value
	(*n* = 210)	(*n* = 273)	(*n* = 260)	
Mother’s age (Mean ± SD)	31.1 ± 0.3	30.4 ± 0.3	29.8 ± 5.2	0.022
Mother’s ethnicity, n (%)				0.031
Chinese	121(57.6)	119 (43.6)	129 (49.6)	
Malay	56(26.7)	88 (32.2)	72 (27.7)	
Indian	33(15.7)	66 ( 24.2)	59 (22.7)	
Mother’s education, n (%)				**0.001**
Below university level	113(53.8)	174(63.7)	185(71.2)	
University level and above	97(46.2)	99(36.3)	75(28.8)	
Marital status, n (%)				0.509
Married	205(97.6)	262(96.0)	249(95.8)	
Not married	5(2.4)	11(4.0)	11(4.2)	
Smoking during pregnancy, n (%)				0.201
Yes	5(2.4)	11(4.0)	4(1.5)	
No	205(97.6)	262(96.0)	256(98.5)	
Mothers BMI (kg/m2) at 26 weeks (mean ± SD)	25.9 ± 0.3	26.7 ± 0.3	26.2 ± 4.4	0.104
Birth order, n (%)				0.069
First child	102(48.6)	104(38.1)	109(41.9)	
Second child or more	108(51.4)	169(61.9)	151(58.1)	
Infant gestational age (Mean ± SD)	38.6 ± 0.07	38.6 ± 0.06	38.5 ± 0.9	0.641
Infant gender				
Female, n (%)	106(50.5)	129(47.3)	121(46.5)	0.669
Infant feeding patterns from 0 up to 6 months of age, n (%)				**0.009**
Any breastfeeding < 4 months	13( 6.2)	10(3.7)	6(2.3)	
Any breastfeeding > 4 months	179(85.2)	215(78.7)	212(81.5)	
Formula fed only	18(8.6)	48(17.6)	42(16.2)	
Infants BMI z-score at different ages (mean ± SD)				
Birth	−0.16(0.09)	0.03(0.08)	−0.15(0.10)	0.896
3 months	−0.14(0.08)	−0.15(0.09)	0.006(0.12)	0.317
6 months	0.03(0.09)	−0.01(0.10)	−0.015(0.13)	0.769
9 months	−0.12(0.09)	−0.18(0.09)	0.06(0.12)	0.332
12 months	−0.13(0.08)	−0.05(0.09)	0.09(0.12)	0.427
15 months	−0.03(0.09)	−0.06(0.09)	0.06(0.12)	0.662
18 months	−0.09(0.09)	−0.15(0.09)	0.04(0.13)	0.331
24 months	−0.05(0.09)	−0.12(0.09)	0.07(0.14)	0.899

*p values were obtained from Chi-squared test for categorical variables and from one-way ANOVA for continuous variables. Statistically significant *p* values < 0.01 were highlighted in bold

### Associations between appetitive traits at 3 and 12 months of age and BMI z-score

Table 2 shows the associations of appetitive traits measured by the BEBQ at 3 months of age with BMI z-score up to 24 months of age. While a positive trend association was found between food responsiveness (BEBQ) and BMI z-score at 3 months (*p* = 0.015), food responsiveness was significantly associated with higher BMI z-scores at age 6 months and up to 15 months (*p* < 0.01). Conversely, slowness in eating and satiety responsiveness was significantly associated with lower BMI z-score at only 6 months of age (*p* = 0.008). A trend of this association was seen again at 15 months of age (*p* = 0.035). No statistically significant associations were seen between enjoyment of food measured at 3 months and BMI z-score (Table 2).

**Table 2:** Multivariate linear regressions of each appetitive trait (independent variables) at 3 months of age measured by the BEBQ on BMI z-score (dependent variables) from 3 months up to 24 months of age

BEBQ appetitive trait subscales						
	Food responsiveness		Slowness in eating and satiety responsiveness		Enjoyment of food	
Age	BMI z-score	^a^Adj.	BMI z-score	^a^Adj.	BMI z-score	^a^Adj.
	β (95 % CI)	*p* value	β (95 % CI)	*p* value	β (95 % CI)	*p* value
3 months	0.15(0.03,0.28)	0.015	−0.11(−0.24,0.02)	0.084	−0.10(−0.22,0.03)	0.120
6 months	**0.24(0.09,0.38)**	**0.001**	**−0.20(−0.35,-0.05)**	**0.008**	−0.02(−0.16,0.13)	0.815
9 months	**0.23(0.09,0.38)**	**0.001**	−0.06(−0.21,0.09)	0.418	0.07(−0.07,0.21)	0.328
12 months	**0.20(0.08,0.32)**	**0.002**	−0.09(−0.22,0.03)	0.151	0.06(−0.06,0.19)	0.325
15 months	**0.17(0.04,0.29)**	**0.009**	−0.14(−0.27,-0.01)	0.035	0.04(−0.09,0.16)	0.590
18 months	0.09(−0.05,0.22)	0.213	−0.12(−0.27,0.02)	0.095	−0.04(−0.19,0.10)	0.546
24 months	0.13(−0.00,0.26)	0.054	−0.07(−0.21,0.07)	0.301	−0.005(−0.14,0.13)	0.938

^*a*^ *p* values adjusted for birth BMI z-score, maternal ethnicity, maternal education, infant feeding patterns up to 6 months of age, mothers age, birth order, smoking during pregnancy, gestational age, pregnancy BMI at 26 weeks. *p* values < 0.01 highlighted in bold are statistically significant. Valid n at 3 months (*n* = 209), 6 months (*n* = 206), 9 months (*n* = 198), 12 months (*n* = 208), 15 months (*n* = 205), 18 months (*n* = 162) and 24 months (*n* = 179)

When examining appetitive traits measured by the CEBQ at age 12 months to the child’s BMI at 12 months up to 24 months of age, no statistically significant associations or trends were seen (*p* > 0.05) (Table 3).

**Table 3:** Multivariate linear regressions of each appetitive trait (independent variables) at 12 months of age measured by the CEBQ on BMI z-score (dependent variables) from 12 months up to 24 months of age

CEBQ appetitive trait subscales								
	Food responsiveness		Slowness in eating		Satiety responsiveness		Enjoyment of food	
Age	BMI z-score	^a^Adj.	BMI z-score	^a^Adj.	BMI z-score	^a^Adj.	BMI z-score	^a^Adj.
	β (95 % CI)	*p* value	β (95 % CI)	*p* value	β (95 % CI)	p value	β (95 % CI)	*P* value
12 months	0.09(−0.03,0.22)	0.150	−0.07(−0.20,0.05)	0.238	−0.07(−0.20,0.05)	0.239	0.07(−0.06,0.19)	0.285
15 months	0.11(0.02,0.23)	0.213	−0.12(−0.25,0.002)	0.054	−0.08(−0.21,0.04)	0.200	0.061(−0.07,0.19)	0.343
18 months	0.01(−0.14,0.16)	0.870	−0.02(−0.11,0.16)	0.759	−0.10(−0.23,0.04)	0.165	0.080(−0.05,0.21)	0.232
24 months	0.07(−0.08,0.21)	0.357	−0.02(−0.15,0.12)	0.808	−0.10(−0.23,0.04)	0.169	0.091(−0.04,0.22)	0.179

^*a*^ *p* values adjusted for birth BMI z-score, maternal ethnicity, maternal education, infant feeding patterns up to 6 months of age, mothers age , birth order, smoking during pregnancy, gestational age, pregnancy BMI at 26 weeks. *p* values < 0.01 highlighted in bold are statistically significant. Valid n at 12 months (*n* = 208), 15 months (*n* = 205), 18 months (*n* = 162), and 24 months (*n* = 179)

Similar results were seen when sensitivity analysis was conducted on all the subjects who responded to the BEBQ (*n* = 403). Food responsiveness (BEBQ) remained significantly associated to BMI z-score from 3 to 15 months of age (*p* < 0.01), while slowness in eating and satiety responsiveness was still negatively associated to BMI z-score at 6 months (*p* = 0.009) (Additional file 3: Table S3). In all the subjects who responded to the CEBQ (*n* = 320), no statistically significant associations or trends were seen between all the appetitive trait subscales to BMI z-scores from 12 to 24 months of age (Additional file 4: Table S4).

### Associations between appetitive traits at 3 and 12 months of age and weight gain

Conditional BMI z-score change, indicating disproportionate weight gain was assessed during the first 2 years. Between 3 to 6 months of age, there were trends of food responsiveness at 3 months of age (BEBQ) being positively associated with greater conditional BMI z-score change (*p* = 0.012), and slowness in eating and satiety responsiveness (BEBQ) being negatively associated with weight gain in the same period (*p* = 0.034). Enjoyment of food (BEBQ) was not associated with weight gain from 3 months up to 24 months of age (Table 4).

**Table 4:** Multivariate linear regressions of each appetitive trait (independent variables) at 3 months of age measured by the BEBQ on conditional BMI z-score change (dependent variables) from 3 months up to 24 months of age

BEBQ appetitive trait subscales						
	Food responsiveness		Slowness in eating and satiety responsiveness		Enjoyment of food	
Age	Conditional	^a^Adj. *P* value	Conditional	^a^Adj. *P* value	Conditional	^a^Adj. *P* value
	BMI z-score change		BMI z-score change		BMI z-score change	
	β (95 % CI)		β (95 % CI)		β (95 % CI)	
3_6 months	**0.18(0.04,0.32)**	**0.012**	**−0.15(−0.29,-0.01)**	**0.034**	0.06(−0.07,0.20)	0.369
6_9 months	0.09(−0.05,0.23)	0.223	0.09(−0.06,0.24)	0.239	0.08(−0.06,0.23)	0.263
9_12 months	0.04(−0.09,0.17)	0.560	−0.06(−0.20,0.17)	0.366	−0.01(−0.13,0.13)	0.979
12_15 months	0.03(−0.10,0.17)	0.627	−0.10(−0.24,0.04)	0.158	−0.02(−0.15,0.19)	0.810
15_18 months	0.02(0.20,0.15)	0.782	−0.07(−0.26,0.11)	0.436	−0.17(−0.35,0.10)	0.054
18_24 months	0.07(−0.07,0.23)	0.378	0.06(−0.11,0.23)	0.485	0.13(−0.05,0.30)	0.153

^*a*^ *p* values adjusted for maternal ethnicity, maternal education, infant feeding patterns up to 6 months of age, mothers age, birth order, smoking during pregnancy, gestational age, pregnancy BMI at 26 weeks. *p* values *p* < 0.01 highlighted in bold are statistically significant. Valid n at 3_6 months (*n* = 200), 6_9 months (*n* = 194), 9_12 months (*n* = 198), 12_15 months (*n* = 204), 15_18 months (*n* = 159), 18_24 months (*n* = 144)

None of the appetitive traits at 12 months of age (CEBQ) had statistically significant associations or trends with conditional BMI z-score change between ages 12 and 24 months (Table 5).

**Table 5:** Multivariate linear regressions of each appetitive trait (independent variable) at 12 months of age measured by the CEBQ on conditional BMI z-score change (dependent variable) from 12 up to 24 months of age

CEBQ appetitive trait subscales								
	Food responsiveness		Slowness in eating		Satiety responsiveness		Enjoyment of food	
Age	Conditional BMI z-score	^a^Adj.	Conditional BMI z-score	^q^Adj.	Conditional BMI z-score	^a^Adj.	Conditional BMI z-score	^a^Adj.
	β (95 % CI)	*p* value	β (95 % CI)	*p* value	β (95 % CI)	p value	β (95 % CI)	*P* value
12_15 months	0.04(−0.10,0.18)	0.605	−0.11(−0.24,0.03)	0.120	−0.01(−0.12,0.15)	0.851	−0.04(−0.17,0.10)	0.589
15_18 months	−0.07(−0.25,0.11)	0.451	0.15(−0.02,0.31)	0.085	−0.04(−0.12,0.21)	0.606	0.01(−0.17,0.17)	0.993
18_24 months	−0.11(−0.07,0.29)	0.987	0.06(−0.22,0.09)	0.428	0.07(−0.08,0.22)	0.374	−0.04(−0.21,0.12)	0.605

^a^ *p* values adjusted for maternal ethnicity, maternal education, infant feeding patterns up to 6 months of age, mothers age, birth order, smoking during pregnancy, gestational age, pregnancy BMI at 26 weeks. *p* values *p* < 0.01 highlighted in bold are statistically significant. Valid n at 12_15 months (*n* = 204), 15_18 months (*n* = 159), 18_24 months (n-144)

The sensitivity analyses showed similar trend associations to weight gain. Food responsiveness (BEBQ) remained positively associated to weight gain between 3 to 6 months of age (*p* = 0.049) and slowness in eating (BEBQ) was still associated to negative weight gain within the same period (*p* = 0.018) (Additional file 5: Table S5). The subjects who responded to the CEBQ (*n* = 320) did not show any statistically significant associations or trends between the appetitive trait subscales to weight gain from 12 to 24 months of age (Additional file 6: Table S6).

## Discussion

In our longitudinal cohort study on early childhood appetitive traits, food responsiveness (a food approach appetitive trait) assessed at 3 months of age was prospectively associated with higher BMI z-scores up to 15 months of age. In contrast, food avoidant appetitive traits like slowness in eating and satiety responsiveness were associated with lower BMI z-scores at an early age of 6 months. However, none of the similar appetitive traits measured at 12 months of age were associated with BMI z-scores in the first two years of life.

Greater food responsiveness assessed at 3 months of age was associated with higher BMI z-score at repeated assessments from 3 until 15 months of age in our study. This suggests that infants who are more responsive to milk feeding cues tend to be heavier, and remain heavier at least up to 15 months, reflective in higher BMI z-score. In contrast, slowness in eating and satiety responsiveness, which is a reflection of the speed an infant typically feeds and an infant’s satiety sensitivity, was inversely associated with BMI z-score at age 6 months and 15 months. Our findings support results from the Gemini cohort study in the United Kingdom which reported positive associations between an infant’s food responsiveness [24, 25] at 3 months of age with weight at 9 months of age, as well as an inverse association of slowness in eating at 3 months with weight at 9 months of age [24]. However, these studies [24, 25] were limited as they only included twins, whose weight and growth in utero and infancy differs from singletons [39]. With this study, we show that these associations are also applicable to term singleton infants within the normal birth weight range.

Higher food responsiveness scores and lower slowness in eating and satiety responsiveness scores were also associated with greater weight gain between 3 to 6 months of age. This indicates that during early infancy, infants who are more responsive to milk cues and feed faster and are less easily satiated tend to have increased weight gain. Our results are again in line with the Gemini study which reported an association of food responsiveness and slowness in eating to weight gain [24, 25]. The importance of when a child first starts gaining weight or becoming overweight was highlighted by the Avon Longitudinal Study of Parents And Children (ALSPAC). This birth cohort study reported that weight gain, particularly in the first year of life leads to increased risk for obesity in childhood [40]. Furthermore, a study on African American young adults reported that participants with rapid weight gain within the first 4 months of life were at higher risk for obesity at 20 years of age [41].

In addition to studying infant appetitive traits at 3 months, we have also chosen to use the CEBQ as a measure of appetitive traits at 12 months to ascertain the eating behaviors of children on solid food early in life. To our knowledge, the only other study to use the CEBQ in children aged 12 months is the study by Svensson et al. 2011, who reported no associations between early appetitive traits and BMI in children aged between 1–6 years of age [9]. Similarly, our study found no statistical significance or obvious trend between the appetitive traits measured at 12 months to BMI z-scores up to 24 months of age. Interestingly, a study by Wright et al. used another measure of appetite (eating avidity) at 12 months age and also found no association with adiposity (using an index calculated from anthropometric, skinfold and bioimpedance data) at 7 years of age [19].

To our knowledge, this is the first study to examine the use of both BEBQ and CEBQ as appetitive traits measures at different time points in the same cohort of healthy single term born children within the healthy birth weight range. In our study, the CEBQ, compared to the BEBQ, does not appear to be associated with BMI z-score and weight gain in early childhood. There are a few possible explanations for this finding. Firstly, our results show that the greatest weight gain occurs between 3 to 6 months of age, and the CEBQ which was administered at 12 months may be limited in its ability to detect smaller changes in weight or weight gain which occurs after that period. Secondly, studies have shown that there is an age effect for several of the appetitive traits measured from the CEBQ. Food approach behaviors measured by food responsiveness, and enjoyment of food scores were reported to be higher, with these traits being more prominent in one year old children compared to older pre-school children [9], whereas food avoidant behaviors tend to develop only later in older children above the age of 2 years [9, 42]. It is possible that at the young age of 12 months certain appetitive traits have not been well established in the children. Consequently, some items in the questionnaire may be less applicable and may thus affect the overall responses to the questionnaire. Thirdly, with more recent evidence showing the lack of association between infant appetitive traits measured at one year of age to weight, weight gain and adiposity in later childhood [9, 19] it is possible that infant appetite traits measured at 3 months may have the greatest impact on weight and adiposity only in the first 2 years of life, and the persistent influence of these appetitive traits to weight gain in later childhood and adult obesity may not be as relevant [18, 19].

### Strengths and limitations

As noted earlier, this is the first study that has compared the use of both the BEBQ and CEBQ in the same cohort to study the association of early appetitive traits at 3 months and 12 months of age with BMI and weight gain tracked longitudinally from 3 months up till 24 months of age. Furthermore, the use of conditional BMI z-score change as a measure of weight gain is an analytical approach that accounts for the strong correlation between BMI z–scores across time points [43].

Limitations of this study need to be acknowledged. Firstly, for the cross-sectional associations of appetitive traits at 3 months with BMI z-score at 3 months of age and appetitive traits at 12 months with BMI z-score at 12 months of age, the possibility of reverse causation cannot be ruled out. Appetitive traits can differ between obese and normal-weight children, where food responsiveness is positively associated with weight, but heavier toddlers could also be viewed by their mothers as being more responsive to food or being a faster eater [12, 13, 40]. However, the majority of these studies observing this relationship was cross-sectional and could not definitively establish that appetitive traits influenced weight gain without excluding the possibility of reverse causality. More bi-directional prospective association studies such as the one reported by van Jaarsveld et al. [24] are required to confirm the direction of the relationship between infant appetitive traits and increased weight/weight gain. Secondly, due to missing values in the BEBQ, CEBQ and outcome variables, only a subset of the total cohort could be included in the current analysis. Although this may have reduced our statistical power for detecting significant associations, our sensitivity analysis based on the group of subjects who responded to either questionnaire showed similar results, suggesting that our results are robust. Thirdly, women who were responders to the questionnaires were more likely to be highly educated and breastfeeding, which may limit generalizability to women with low education and those who are formula feeding. Lastly, we acknowledge that both the BEBQ and CEBQ are parent-reported questionnaires and are thus based on parental perception and interpretation of child eating behavior. Empirical data from experimental studies observing actual eating behavior of children is needed to further understand how accurate parent –reported behaviors reflect that of actual food responses in a child.

## Conclusion

This study provides evidence to support the notion that early appetitive traits in infancy - specifically appetitive traits of food responsiveness predict higher BMI z-scores and greater weight gain in healthy term born children. Appetitive traits measured at 3 months but not 12 months was associated with a child’s BMI z-score and weight gain in the first 24 months after birth. Although CEBQ is a valuable psychometric instrument, adjustments to some of the question items might be required if administered at a younger age of 12 months. Future qualitative studies are warranted to understand the applicability of the CEBQ at 12 months of age.

## Additional files

Additional file 1: Table S1. Factor loadings for all items of the Baby Eating Behavior Questionnaire (BEBQ) and Cronbach alpha scores for each factor structure (DOCX 17 kb) Additional file 2: Table S2. Factor loadings for all items of the Children Eating Behavior Questionnaire (CEBQ) and Cronbach alpha scores for each factor structure (DOCX 19 kb) Additional file 3: Table S3. Multivariate linear regressions of each appetitive trait (independent variable) at 3 months of age measured by the BEBQ on BMI z-score (dependent variable) from 3 months up to 24 months of age in all the subjects that answered the BEBQ (*n* = 403) (DOCX 16 kb) Additional file 4: Table S4. Multivariate linear regressions of each appetitive trait ( independent variable) at 12 months of age measured by the CEBQ on BMI z-score (dependent variable) from 12 months up to 24 months of age in all the subjects that answered the CEBQ (*n* = 320) (DOCX 16 kb) Additional file 5: Table S5. Multivariate linear regressions of each appetitive trait ( independent variable) at 3 months of age measured by the BEBQ on conditional BMI z-score change (dependent variable) from 3 months up to 24 months of age in all the subjects that answered the BEBQ (*n* = 403) (DOCX 16 kb) Additional file 6: Table S6. Multivariate linear regressions of each appetitive trait ( independent variable) at 12 months of age measured by the CEBQ on conditional BMI z-score change ( dependent variable) from 12 up to 24 months of age in all the subjects that answered the CEBQ (*n* = 320) (DOCX 16 kb)

## Abbreviations

BEBQ: Baby Eating Behavior Questionnaire
CEBQ: Child Eating Behavior Questionnaire
BMI: Body mass index
