# Supplementary material for: Prospective associations of appetitive traits at 3 and 12 months of age with body mass index and weight gain in the first 2 years of life
Source: BMC Pediatr. 2015 Oct 12;15:153. doi: 10.1186/s12887-015-0467-8 (PMC4603814; doi:10.1186/s12887-015-0467-8)
Supplement: Additional file 1: Table S1. — Factor loadings for all items of the Baby Eating Behavior Questionnaire (BEBQ) and Cronbach alpha scores for each factor structure (DOCX 17 kb) [file 12887_2015_467_MOESM1_ESM.docx]

Supplementary Table 1: Factor loadings for all items of the Baby Eating Behavior Questionnaire (BEBQ) and Cronbach alpha scores for each factor structure.

| Items ^a^ | Factors determined through factor analysis ^b^ | | | | Original scale ^d^ | Cronbach alpha |
| --- | --- | --- | --- | --- | --- | --- |
|  | 1  ‘ Food Responsiveness’ | 2 ‘ Slowness in eating’/ ‘ Satiety Responsiveness’ | 3  ’Enjoyment of food’ | 4^c^  ‘ Not applicable’ |  |  |
| My baby frequently wants more milk than I can provide | 0.609 |  |  |  | FR | 0.796 |
| My baby has a big appetite | 0.628 |  |  |  | GA |  |
| If allowed to, my baby would take too much milk | 0.706 |  |  |  | FR |  |
| Even when my baby has just eaten well, he/she is happy to feed again if offered | 0.759 |  |  |  | FR |  |
| My baby is always demanding a feed | 0.684 |  |  |  | FR |  |
| If given the chance, my baby would always be feeding | 0.769 |  |  |  | FR |  |
|  |  |  |  |  |  |  |
| My baby finishes feeding quickly (R) |  | 0.564 |  |  | SE | 0.767 |
| My baby takes more than 30 minutes to finish feeding |  | 0.782 |  |  | SE |  |
| My baby gets full before taking all the milk I think he/she should have |  | 0.598 |  |  | SR |  |
| My baby feeds slowly |  | 0.807 |  |  | SE |  |
| My baby finds it difficult to manage a complete feed |  | 0.640 |  |  | SR |  |
|  |  |  |  |  |  |  |
|  |  |  |  |  |  |  |
| My baby seems contented while feeding |  |  | 0.701 |  | EF | 0.600 |
| My baby loves milk |  |  | 0.733 |  | EF |  |
| My baby enjoys feeding time |  |  | 0.653 |  | EF |  |
|  |  |  |  |  |  |  |
| My baby becomes distressed while feeding (R)  My baby gets full up easily  My baby sucks more and more slowly during the course of a feed |  |  |  | 0.672  0.638    0.510 | EF  SR    SE | 0.401 |
|  |  |  |  |  |  |  |

^a^ Items marked with (R) have been reversed scored. There are only 17 items in the table as the item ‘My baby can easily take a feed within 30 minutes of the last one’ had a factor loading score below 0.5 and was not included into the subscales.

^b^ Only items with factor loading scores above 0.5 are presented

^c^ The fourth subscale was labelled as ‘ non applicable’ as items do not load well this subscale and it was not used for further analysis

^d^ Appetite scale the items was originally intended to measure: EF,’ enjoyment of food’ ; FR, ‘ food responsiveness’; GA,’ general appetite’ ; SR ‘ satiety responsiveness’
