# Supplementary material for: Prospective associations of appetitive traits at 3 and 12 months of age with body mass index and weight gain in the first 2 years of life
Source: BMC Pediatr. 2015 Oct 12;15:153. doi: 10.1186/s12887-015-0467-8 (PMC4603814; doi:10.1186/s12887-015-0467-8)
Supplement: Additional file 2: Table S2. — Factor loadings for all items of the Children Eating Behavior Questionnaire (CEBQ) and Cronbach alpha scores for each factor structure (DOCX 19 kb) [file 12887_2015_467_MOESM2_ESM.docx]

Supplementary Table 2: Factor loadings for all items of the Children Eating Behavior Questionnaire (CEBQ) and Cronbach alpha scores for each factor structure.

| Items ^a^ | Factors determined through factor analysis ^b^ | | | | | | |  | Original scale ^d^ | Cronbach alpha |
| --- | --- | --- | --- | --- | --- | --- | --- | --- | --- | --- |
|  | 1  ‘Enjoyment of food/ Food fussiness | 2  ‘Emotional under eating’ | 3  ‘Emotional over eating’ | 4  ‘Slowness in eating’ | 5  ‘Food responsiveness’ | 6  ‘Desire to drink’ | 7  ’Satiety responsiveness’ | 8  ‘ Not applicable ^c^’ |  |  |
| My child loves food | 0.788 |  |  |  |  |  |  |  | EF | 0.909 |
| My child is interested in food  My child refuses new foods at first(R) | 0.826  0.633 |  |  |  |  |  |  |  | EF  FF |  |
| My child enjoys tasting new foods  My child enjoys a wide variety of foods  My child looks forward to mealtimes | 0.767  0.798  0.671 |  |  |  |  |  |  |  | FF  FF  EF |  |
| My child enjoys eating | 0.751 |  |  |  |  |  |  |  | EF |  |
| My child is difficult to please with meals (R) | 0.602 |  |  |  |  |  |  |  | FF |  |
| My child is interested in tasting food s/he hasn't tasted before | 0.688 |  |  |  |  |  |  |  | FF |  |
| My child decides that s/he doesn't like a food, even without tasting it (R)  My child eats less when angry  My child eats less when s/he is tired  My child eats more when she is happy  My child eats less when upset | 0.592 | .  0.729  0.784  0.577  0.867 |  |  |  |  |  |  | FF  EUE  EUE  EUE  EUE | 0.785 |
|  |  |  |  |  |  |  |  |  |  |  |
|  |  |  |  |  |  |  |  |  |  |  |
| My child eats more when worried  My child eats more when annoyed  My child eats more when anxious |  |  | 0.762  0.774  0.787 |  |  |  |  |  | EOE  EOE  EOE | 0.778 |
|  |  |  |  |  |  |  |  |  |  |  |
| My child finishes his/her meal quickly  My child eats slowly  My child takes more than 30 minutes to finish a meal |  |  |  | 0.743  0.768  0.599 |  |  |  |  | SE  SE  SE | 0.753 |
|  |  |  |  |  |  |  |  |  |  |  |
|  |  |  |  |  |  |  |  |  |  |  |
| If allowed to, my child would eat too much  Even if my child is full up s/he finds room to eat his/her favorite food  If given the chance, my child would always have food in his/her mouth |  |  |  | 0.583    0.712    0.585 |  |  |  |  | FR    FR    FR | 0.777 |
|  |  |  |  |  |  |  |  |  |  |  |
| My child is always asking for a drink  If given the chance, my child would drink continuously throughout the day  If given the chance, my child would always be having a drink |  |  |  |  | 0.746  0.758  0.829 |  |  |  | DD  DD  DD | 0.739 |
|  |  |  |  |  |  |  |  |  |  |  |
| My child leaves food on his/her plate at the end of a meal  My child gets full before his/her meal is finished  My child gets full up easily |  |  |  |  |  | 0.627  0.807  0.527 |  |  | SR  SR  SR | 0.683 |
|  |  |  |  |  |  |  |  |  |  |  |
| My child is always asking for food |  |  |  |  |  |  |  | 0.513 | SR |  |
|  |  |  |  |  |  |  |  |  |  |  |
|  |  |  |  |  |  |  |  |  |  |  |

a Items marked with (R) have been reversed scored. There are only 30 items in the table as the item ‘My child has a big appetite’, Given the choice, my child would eat most of the time, ‘My child eats more s/he has nothing else to do’, ‘My child cannot eat a meal if s/he has had a snack just before’, ‘My child eats more and more slowly during the course of a meal’ had a factor loading score below 0.5 and was not included into the subscales.

b Only items with factor loading scores above 0.5 are presented

c The eighth subscale was labelled as ‘ non applicable’ as only one item loaded in this subscale and it was not used for further analysis

d Appetite scale the item was originally intended to measure: EF,’ enjoyment of food’ ; FR, ‘ food responsiveness’, FF,’ food fussiness’ ,SR ‘ satiety responsiveness’; SE ‘slowness in eating’ ; EUE, ‘ Emotional Under Eating’ ; EOE, ‘ Emotional Over Eating’ ; DD, ‘desire to drink’
