# Supplementary material for: Prospective associations of appetitive traits at 3 and 12 months of age with body mass index and weight gain in the first 2 years of life
Source: BMC Pediatr. 2015 Oct 12;15:153. doi: 10.1186/s12887-015-0467-8 (PMC4603814; doi:10.1186/s12887-015-0467-8)
Supplement: Additional file 3: Table S3. — Multivariate linear regressions of each appetitive trait (independent variable) at 3 months of age measured by the BEBQ on BMI z-score (dependent variable) from 3 months up to 24 months of age in all the subjects that answered the BEBQ (n = 403) (DOCX 16 kb) [file 12887_2015_467_MOESM3_ESM.docx]

Supplementary Table 3:Multivariate linear regressions of each appetitive trait (independent variable) at 3 months of age measured by the BEBQ on BMI z-score (dependent variable) from 3 months up to 24 months of age in all the subjects that answered the BEBQ (n=403).

| BEBQ appetitive trait subscales | | | | | | | | |
| --- | --- | --- | --- | --- | --- | --- | --- | --- |
| Food responsiveness | | |  | Slowness in eating and satiety responsiveness | |  | Enjoyment of food | |
|  |  |  |  |  |  |  |  |  |
| Age | BMI z-score | ^a^ Adj. |  | BMI z-score | ^a^Adj. |  | BMI z-score | ^a^Adj. |
|  | β (95%CI) | *p* value |  | β (95%CI) | *p* value |  | β (95%CI) | *p* value |
|  |  |  |  |  |  |  |  |  |
| 3 months | **0.21(0.08,0.34)** | **0.002** |  | -0.09(-0.22,0.04) | 0.187 |  | -0.10(-0.22,0.03) | 0.149 |
| 6 months | **0.25(0.11,0.39)** | **0.001** |  | **-0.20(-0.34,-0.05)** | **0.009** |  | -0.02(-0.16,0.13) | 0.813 |
| 9 months | **0.23(0.09,0.370)** | **0.001** |  | -0.08(-0.23,0.07) | 0.296 |  | 0.08(-0.06,0.22) | 0.275 |
| 12 months | **0.19(0.07,0.31)** | **0.002** |  | -0.11(-0.24,0.02) | 0.083 |  | 0.07(-0.05,0.19) | 0.262 |
| 15 months | **0.15(0.03,0.28)** | **0.009** |  | -0.16(-0.22,-0.03) | 0.016 |  | 0.04(-0.09,0.17) | 0.513 |
| 18 months | 0.06(-0.07,0.20) | 0.361 |  | -0.13(-0.27,0.03) | 0.097 |  | -0.06(-0.21,0.08) | 0.546 |
| 24 months | 0.12(-0.01,0.25) | 0.072 |  | -0.08(-0.22,0.05) | 0.231 |  | -0.01(-0.14,0.13) | 0.957 |

*^a^ p* values adjusted for birth BMI z-score, maternal ethnicity, maternal education, infant feeding patterns up to 6 months of age, mothers age, birth order, smoking during pregnancy, gestational age, pregnancy BMI at 26 weeks. *p* values <0.01 highlighted in bold are statistically significant. Valid n at 3 months (n=402), 6 months (n=375), 9 months (n=359), 12 months (n=369), 15 months (n=366), 18 months (n=295) and 24 months n= (316).
