# Supplementary material for: Prospective associations of appetitive traits at 3 and 12 months of age with body mass index and weight gain in the first 2 years of life
Source: BMC Pediatr. 2015 Oct 12;15:153. doi: 10.1186/s12887-015-0467-8 (PMC4603814; doi:10.1186/s12887-015-0467-8)
Supplement: Additional file 4: Table S4. — Multivariate linear regressions of each appetitive trait ( independent variable) at 12 months of age measured by the CEBQ on BMI z-score (dependent variable) from 12 months up to 24 months of age in all the subjects that answered the CEBQ (n = 320) (DOCX 16 kb) [file 12887_2015_467_MOESM4_ESM.docx]

Supplementary Table 4: Multivariate linear regressions of each appetitive trait ( independent variable) at 12 months of age measured by the CEBQ on BMI z-score (dependent variable) from 12 months up to 24 months of age in all the subjects that answered the CEBQ (n=320).

| CEBQ appetitive trait subscales | | | | | | | | |  | |  | |
| --- | --- | --- | --- | --- | --- | --- | --- | --- | --- | --- | --- | --- |
|  | Food responsiveness |  |  | Slowness in eating |  |  | Satiety responsiveness |  | Enjoyment of food/ Food fussiness | |  | |
|  |  |  |  |  |  |  |  |  |  |  |  |  |
| Age | BMI z-score | ^a^Adj. |  | BMI z-score | ^q^Adj. |  | BMI z-score | ^a^Adj. | BMI z-score | | | ^a^Adj. |
|  | β (95%CI) | *^p^* ^value^ |  | β (95%CI) | *^p^* ^value^ |  | β (95%CI) | *^p^* ^value^ | β (95%CI) | *^p^* ^value^ | | |
|  |  |  |  |  |  |  |  |  |  |  | | |
| 12 months | 0.06(-0.07,0.19) | 0.278 |  | -0.05(-0.17,0.07) | 0.423 |  | -0.07(-0.19,0.06) | 0.239 | 0.07(-0.06,0.19) | 0.278 | | |
| 15 months | 0.07(-0.07,0.20) | 0.329 |  | -0.10(-0.23,0.03) | 0.054 |  | -0.07(-0.20,0.06) | 0.295 | 0.06(-0.07,0.18) | 0.400 | | |
| 18 months | -0.01(-0.16,0.15) | 0.965 |  | 0.02(-0.12,0.15) | 0.808 |  | -0.07(-0.21,0.07) | 0.165 | 0.06(-0.08,0.19) | 0.413 | | |
| 24months | 0.05(-0.10,0.19) | 0.357 |  | -0.01(-0.14,0.13) | 0.966 |  | -0.08(-0.21,0.06) | 0.262 | 0.08(-0.05,0.21) | 0.228 | | |
|  |  |  |  |  |  |  |  |  |  |  | | |

*^a^p* values adjusted for birth BMI z-score, maternal ethnicity, maternal education, infant feeding patterns up to 6 months of age, mothers age , birth order, smoking during pregnancy, gestational age, pregnancy BMI at 26 weeks. *p* values <0.01 highlighted in bold are statistically significant. Valid n at 12 months (n=208), 15 months (n=205), 18 months (n=162), and 24 months (n=179).
