# Supplementary material for: Prospective associations of appetitive traits at 3 and 12 months of age with body mass index and weight gain in the first 2 years of life
Source: BMC Pediatr. 2015 Oct 12;15:153. doi: 10.1186/s12887-015-0467-8 (PMC4603814; doi:10.1186/s12887-015-0467-8)
Supplement: Additional file 5: Table S5. — Multivariate linear regressions of each appetitive trait ( independent variable) at 3 months of age measured by the BEBQ on conditional BMI z-score change (dependent variable) from 3 months up to 24 months of age in all the subjects that answered the BEBQ (n = 403) (DOCX 16 kb) [file 12887_2015_467_MOESM5_ESM.docx]

Supplementary Table 5: Multivariate linear regressions of each appetitive trait ( independent variable) at 3 months of age measured by the BEBQ on conditional BMI z-score change (dependent variable) from 3 months up to 24 months of age in all the subjects that answered the BEBQ (n=403).

| BEBQ appetitive trait subscales | | | | | | | | | |
| --- | --- | --- | --- | --- | --- | --- | --- | --- | --- |
| Food responsiveness | | | |  | Slowness in eating and satiety responsiveness | |  | Enjoyment of food | |
|  |  |  |  |  |  |  |  |  |  |
| Age | Conditional | ^a^Adj. | |  | Conditional | ^a^Adj. |  | Conditional | ^a^Adj. |
|  | BMI z-score change |  |  |  | BMI z-score change |  |  | BMI z-score change |  |
|  | β (95%CI) | ^P value^ | |  | β (95%CI) | ^P value^ |  | β (95%CI) | ^P value^ |
|  |  | | |  |  |  |  |  |  |
| 3_6 months | 0.14(0.01,0.28) | | 0.049 |  | -0.17(-0.31,-0.03) | 0.018 |  | 0.08(-0.06,0.22) | 0.262 |
| 6_9 months | 0.07(-0.07,0.22) | | 0.319 |  | 0.09(-0.09,0.21) | 0.438 |  | 0.10(-0.05,0.21) | 0.185 |
| 9_12 months | 0.04(-0.09,0.16) | | 0.573 |  | -0.08(-0.22,0.06) | 0.269 |  | -0.01(-0.12,0.13) | 0.946 |
| 12_15 months | 0.02(-0.11,0.15) | | 0.769 |  | -0.11(-0.25,0.03) | 0.112 |  | -0.01(-0.15,0.12) | 0.852 |
| 15_18 months | -0.03(-0.12,0.14) | | 0.729 |  | -0.05(-0.24,0.13) | 0.560 |  | -0.19(-0.36,-0.01) | 0.041 |
| 18_24 months | 0.08(-0.08,0.23) | | 0.338 |  | 0.04(-0.12,0.21) | 0.599 |  | 0.13(-0.04,0.30) | 0.125 |
|  |  | |  |  |  |  |  |  |  |

*^a^ p* values adjusted for maternal ethnicity, maternal education, infant feeding patterns up to 6 months of age, mothers age , birth order, smoking during pregnancy, gestational age, pregnancy BMI at 26 weeks. *p* values *p*<0.01 highlighted in bold are statistically significant. Valid n at 3_6 months (n=375), 6_9 months (n=345), 9_12 months (n=346), 12_15 months (n=353), 15_18 months (n=284), 18_24 months (n=253).
