# Supplementary material for: Prospective associations of appetitive traits at 3 and 12 months of age with body mass index and weight gain in the first 2 years of life
Source: BMC Pediatr. 2015 Oct 12;15:153. doi: 10.1186/s12887-015-0467-8 (PMC4603814; doi:10.1186/s12887-015-0467-8)
Supplement: Additional file 6: Table S6. — Multivariate linear regressions of each appetitive trait ( independent variable) at 12 months of age measured by the CEBQ on conditional BMI z-score change ( dependent variable) from 12 up to 24 months of age in all the subjects that answered the CEBQ (n = 320) (DOCX 16 kb) [file 12887_2015_467_MOESM6_ESM.docx]

Supplementary Table 6: Multivariate linear regressions of each appetitive trait ( independent variable) at 12 months of age measured by the CEBQ on conditional BMI z-score change ( dependent variable) from 12 up to 24 months of age in all the subjects that answered the CEBQ (n=320).

| CEBQ appetitive trait subscales | | | | | | |  |  |  |
| --- | --- | --- | --- | --- | --- | --- | --- | --- | --- |
|  | Food responsiveness |  | Slowness in eating |  | Enjoyment of food |  | Satiety responsiveness |  |  |
|  |  |  |  |  |  |  |  |  |  |
| Age | BMI z-score | ^a^Adj.  *p* value | BMI z-score | ^a^Adj. | BMI z-score | ^a^Adj. | BMI z-score | ^a^Adj. |  |
|  | β (95%CI) |  | β (95%CI) | *p* value | β (95%CI) | *p* value | β (95%CI) | *p* value |  |
|  |  |  |  |  |  |  |  |  |  |
| 12_15 months | -0.01(-0.14,0.14) | $0.896$ | -0.08(-0.22,0.06) | 0.279 | -0.02(-0.16,0.13) | 0.831 | -0.05(-0.19,0.09) | 0.491 |  |
| 15_18 months | -0.08(-0.26,0.10) | 0.389 | -0.06(-0.11,0.23) | 0.493 | -0.03(-0.19,0.14) | 0.904 | 0.01(-0.17,0.17) | 0.966 | |
| 18_24 months | -0.01(-0.16,0.17) | 0.987 | -0.01(-0.14,0.16) | 0.897 | 0.03(-0.12,0.18) | 0.69 | -0.01(-0.16,0.15) | 0.934 | |
|  |  |  |  |  |  |  |  |  | |

^a^ *p* values adjusted for maternal ethnicity, maternal education, infant feeding patterns up to 6 months of age, mothers age , birth order, smoking during pregnancy, gestational age, pregnancy BMI at 26 weeks. *p* values *p*<0.01 highlighted in bold are statistically significant. Valid n at 12_15 months (n=204), 15_18 months (n=159), 18_24 months (n=144).
